# Isolated Fetal Ascites Secondary to Persistent Urogenital Sinus

**DOI:** 10.1155/2009/219010

**Published:** 2009-02-15

**Authors:** D. Camanni, A. Zaccara, M. L. Capitanucci, G. Mosiello, I. Capolupo, B. D. Iacobelli, M. De Gennaro

**Affiliations:** ^1^Urodynamics unit, Department of Nephrology and Urology, Bambino Gesù Children's Hospital, 00165 Rome, Italy; ^2^Department of Neonatology, Bambino Gesù Children's Hospital, 00165 Rome, Italy; ^3^Neonatal Surgery Unit, Medical and Surgical Department of Neonatology, Bambino Gesù Children's Hospital, 00165 Rome, Italy

## Abstract

*Objective*. To present a case of isolated ascites secondary due to urogenital abnormalities (urogenital sinus) without any other prenatal ultrasound marker. *Method*. A 36-year-old woman with prenatal isolated ascites delivered a female baby, weighing 2.285 g; ascites was drained at birth and the baby underwent several episodes of urinary retention prior to undergoing X-ray investigations. *Results*. A voiding cystourethrogram revealed a short urogenital sinus: a vesicostomy was performed. A vaginoscopy revealed double vagina with a large posterior vagina. A posterior sagittal anorectal pull-through with genitoplasty was performed at 2 years old with 1-year follow-up. *Conclusions*. Though rare, a urogenital abnormality is to be suspected in fetal ascites cases with negative viral tests and no cardiac anomalies. The most common ultrasound marker of such abnormalities (fluid filled cavity) may be missing because of complete drainage of urine through the tubes into peritoneum.

## 1. Introduction

Persistent urogenital sinus (UGS) is a rare congenital anomaly in which failure of urethrovaginal separation results in one orifice for bladder and vagina to drain through.

These patients exhibit a great internal and external anatomical variability, which may lead to aberrant drainage of urine because of the confluence of the two systems. In recent years, prenatal diagnosis has enabled identification of this condition early in gestation. The commonest ultrasound finding is the presence of a cystic pelvic structure with or without septa, which represents a fluid-filled vagina and which may compress urinary tract (hydrometrocolpos) [1].

Occasionally, some cases may also present with prenatally detected urinary ascites due to urinary reflux through the genital system into peritoneum. We herein report the case of persistent UGS in which prenatal ascites was the only ultrasonographic finding.

## 2. Case Report

**Figure 1:**
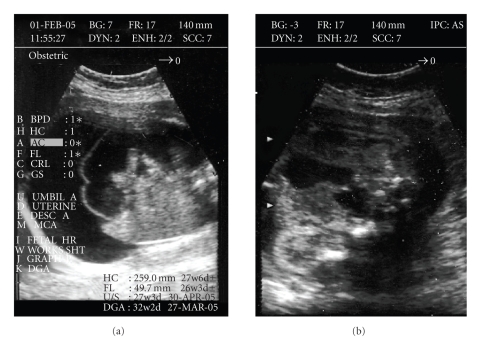
(a) Prenatal ultrasound of the fetus with significant ascites. (b) Moderately distended bladder is visible in a pelvic scan.

A 36-year-old woman, gravida 3, para 2, was presented at 22-week gestation with ultrasonographic evidence of fetal ascites. Amniotic fluid was normal and TORCH serology was negative. Fetal anatomy was unremarkable with the exception of a moderately distended bladder (Figures 1(a), 1(b)). Amniocentesis for karyotyping showed 46, XX and ascites was confirmed at 31-week gestation with significant elevation of diaphragm, prompting elective caesarean section.

A 2.285 g female newborn was delivered. A physical examination revealed a massively distended abdomen with normal looking external genitalia. Four hundred millilitres of clear ascitic fluid were drained from abdominal cavity with immediate improving of respiratory status. Fluid analysis revealed some nucleated cells with normal protein concentration. On day 2 of life, a distended bladder became evident; Foley catheter was positioned without difficulty, which drained normal urine. Renal ultrasound did not show any urinary tract dilatation. Over the following days, an incremental diuresis was observed with no ascites reaccumulation.

**Figure 2:**
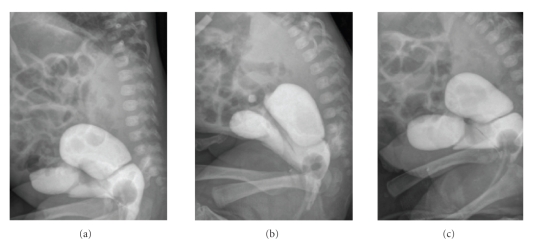
Voiding cystogram showing UGS with Foley catheter at the confluence of bladder and vagina.

After removal of bladder catheter, bladder distended again and the catheter had to be reinserted. A voiding cystogram was then performed which demonstrated a short (1.5 cm) UGS with the catheter at the junction between bladder and vagina (Figure 2). UGS was confirmed at cystoscopy and vesicostomy was carried out since baby was persistently unable to void. At vaginoscopy, a duplicated vagina draining into sinus was found. At 2 years, the baby underwent urogenital reconstruction with total urogenital mobilization. At 2-year follow-up, the patient has complete day-time urinary continence with some residual wet episodes in the night.

## 3. Discussion

Persistent UGS is considered secondary to failure of normal descent of the urogenital septum, leaving confluence of vagina and urethra proximal to the introitus. Such condition is most commonly associated to adrenogenital syndrome with various degrees of virilisation [2] but it may also occur in normal appearing external genitalia, as it was in our patient.

As previously mentioned, distended vagina with hydrometrocolpos was the most common antenatal finding [3]. As Adams et al. pointed out [4], this may be due to significant outflow resistance to urine through the common channel which may impede good emptying of urine. Hydrometrocolpos may be the result of backflow into the vagina during micturition [5]. Ascites, in turn, can be secondary to urine reflux of urine through fallopian tubes. In an extensive review of nonimmune fetal ascites, Favre et al. reported ascites of urinary origin in 15 cases. Posterior urethral valves were diagnosed in 14 cases and ureterocele in one. All cases presented with remarkable dilatation of urinary tract [6]. These authors also reported prenatal ascites in 8 cases of cloacal dysgenesis; however, this was never an isolated finding.

In 2008, Puhl et al. described fetal urogenital sinus with associated urogenital defects (bilateral hydronephrosis). Ascites was moderate [7], the same findings were confirmed by Gul et al. who reported association of UGS, ureterocele, hydroureter, and hydronephrosis [8].

Our patient was at significant variance with previously reported cases in that isolated ascites was the only documented finding throughout pregnancy.

Therefore, attention was primarily directed to common causes of isolated ascites such as viral infections or cardiovascular problems [9, 10]. As previously mentioned, TORCH serology and all other examinations were normal both antenatally and immediately after birth. However, because of significant elevation of diaphragm, ascites had to be drained before establishing diagnosis. It was not until the second day of life when poor bladder drainage demanded catheterization, that a urogenital problem was suspected.

We do not have an explanation for the absence of any vaginal distention at prenatal ultrasound. We might speculate that a free flow of urine was present at uterovaginal junction preventing fluid accumulation; however, we have not been able to confirm such hypothesis intraoperatively neither there is any study in Literature describing anatomical features of uterovaginal junction in patients with UGS or cloacal anomalies.

Moreover, in our patient, the UGS was only 1.5 cm in length and this makes interpretation of prenatal ultrasound findings even more difficult. As a matter of fact, intraperitoneal reflux of urine in UGS patients was observed only in long, narrow sinuses with high confluence or vagina [11].

## 4. Conclusions

Our findings suggest that prenatal diagnosis of persistent UGS should be suspected not only in cases of documented hydrocolpos but also in cases of isolated ascites. The underlying mechanism of such accumulation may be secondary to reflux of urine into peritoneum. Viral tests and screening for cardiac abnormalities should continue to be first line examinations; however, in case of negative tests and unremarkable fetal anatomy, the suspicion of urogenital anomalies should be incorporated into prenatal counselling with appropriate information about such conditions being conveyed to the prospective parents.
